# Isolation of a multipotent mesenchymal stem cell-like population from human adrenal cortex

**DOI:** 10.1530/EC-18-0067

**Published:** 2018-04-05

**Authors:** Earn H Gan, Wendy Robson, Peter Murphy, Robert Pickard, Simon Pearce, Rachel Oldershaw

**Affiliations:** 1 Institute of Genetic MedicineNewcastle University, International Centre for Life, Central Parkway, Newcastle upon Tyne, UK; 2 Endocrine UnitRoyal Victoria Infirmary, Newcastle upon Tyne, UK; 3 Urology UnitFreeman Hospital, Newcastle upon Tyne, UK; 4 Institute of Cellular MedicineNewcastle University, Newcastle upon Tyne, UK; 5 Department of Musculoskeletal BiologyInstitute of Ageing and Chronic disease, University of Liverpool, Liverpool, UK

**Keywords:** mesenchymal stem cells, adrenocortical stem cell, tissue regeneration, autoimmune Addison’s disease

## Abstract

**Background:**

The highly plastic nature of adrenal cortex suggests the presence of adrenocortical stem cells (ACSC), but the exact *in vivo* identity of ACSC remains elusive. A few studies have demonstrated the differentiation of adipose or bone marrow-derived mesenchymal stem cells (MSC) into steroid-producing cells. We therefore investigated the isolation of multipotent MSC from human adrenal cortex.

**Methods:**

Human adrenals were obtained as discarded surgical material. Single-cell suspensions from human adrenal cortex (*n* = 3) were cultured onto either complete growth medium (CM) or MSC growth promotion medium (MGPM) in hypoxic condition. Following *ex vivo* expansion, their multilineage differentiation capacity was evaluated. Phenotype markers were analysed by immunocytochemistry and flow cytometry for cell-surface antigens associated with bone marrow MSCs and adrenocortical-specific phenotype. Expression of mRNAs for pluripotency markers was assessed by q-PCR.

**Results:**

The formation of colony-forming unit fibroblasts comprising adherent cells with fibroblast-like morphology were observed from the monolayer cell culture, in both CM and MGPM. Cells derived from MGPM revealed differentiation towards osteogenic and adipogenic cell lineages. These cells expressed cell-surface MSC markers (CD44, CD90, CD105 and CD166) but did not express the haematopoietic, lymphocytic or HLA-DR markers. Flow cytometry demonstrated significantly higher expression of GLI1 in cell population harvested from MGPM, which were highly proliferative. They also exhibited increased expression of the pluripotency markers.

**Conclusion:**

Our study demonstrates that human adrenal cortex harbours a mesenchymal stem cell-like population. Understanding the cell biology of adrenal cortex- derived MSCs will inform regenerative medicine approaches in autoimmune Addison’s disease.

## Introduction

The plasticity of adrenal cortex has been shown to confer a therapeutic potential for immunomodulatory and regenerative therapy in adrenal insufficiency (1, 2, 3). However, despite the demonstration of the regenerative capacity of rodent adrenal capsule nearly 80 years ago (4), the exact *in vivo* identity of adrenocortical stem cells (ACSCs) remains elusive. Adult mesenchymal stromal or stem cells (MSCs) have drawn significant interest among researchers in the stem cell field, owing to their multipotent differentiation capacity, low tumorigenicity and tolerogenic nature for allogenic cell-based therapies. They were initially isolated from the adult bone marrow and have subsequently been harvested from several other tissues, including the adipose tissue (5), pancreas (6), umbilical cord (7), synovium (8), dental pulp (9), trabeculae bone (10), peripheral blood (11) and skeletal muscle (12, 13).

MSCs lack a unique and specific surface antigen that can be used for positive selection. Hence, the characteristics of bone marrow–derived MSCs are commonly used as the ‘gold standard’ to define MSCs derived from other tissues. Bone marrow–derived MSCs exhibit the ability to adhere to plastic dishes in a standard culture condition and express a set of phenotypic markers on their surface, including CD44, CD90, CD105 and CD166 (14, 15). They appear first as adherent, single colony clusters (colony-forming unit fibroblasts CFU-F) before growing as a homogenous population of adherent cells on culture dishes (16). They also have the capacity to differentiate along mesodermal lineages into osteocytes, chondrocytes and adipocytes (14, 15), following specific *in vitro* culture condition and supplementation with exogenous soluble factors (17).

Although the exact *in vivo* identity of ACSCs is yet to be defined in either rodents or humans, they are thought to reside in the capsular and subcapsular regions of the adrenal cortex. A few important transcription factors and signalling pathways (e.g. steroidogenic factor 1 (SF1), sonic hedgehog signalling pathway (SHH-GLI)) have been identified as being crucial in the maintenance and regulation of ACSCs (18). Conceptually, MSCs are the postnatal progenitor cells of most derivatives of mesoderm (13) and the adrenal gland originates from the intermediate mesoderm embryonically. Therefore, adrenocortical progenitor cells are likely derived from MSC or a closely allied cell-type. In recent years, a few studies have demonstrated that adenovirus-mediated forced expression of *SF1* could transform rodent and human adipose tissue or bone marrow-derived MSCs into steroidogenic cells, with the ability to produce multiple steroid hormones in response to adrenocorticotropic hormone (19, 20, 21, 22, 23). This finding suggests that MSCs represent a potential source of stem cells for producing steroidogenic cells. Hence, we investigated the direct isolation and characterisation of MSCs from human adrenal cortex, which could potentially be the previously uncharacterised ACSC.

## Materials and methods

### Primary cell culture of human adrenal cortical cells

Adult adrenal tissue was obtained with written consent from patients undergoing radical nephrectomy for upper pole renal cell carcinoma, where the planned surgery meant that the adrenal gland would have to be sacrificed. The study was approved by the National Research Ethics Service Committee North East-Sunderland Research Ethics Committee (12/NE/0101). The adrenal cortical tissue was separated from fat and the adrenal medulla by removing tissue adjacent to the central vein. Adrenal cortex with intact capsule was then minced and enzymatically dispersed for 30 min in a digestive solution comprising 0.2% collagenase (2 mg/mL) (Sigma) and 0.01% deoxyribonuclease I (DNAse I) (0.1 mg/mL) (Sigma), at 37°C. The digested tissues were then disaggregated and filtered through a 70 µm nylon cell strainer. The undigested tissue fragments were resubmitted to the same digestion procedure until all tissues were fully digested. The filtered cells were centrifuged and re-suspended in two types of growth media. Half of the adrenocortical cells were seeded in a complete growth medium (CM), comprising DMEM: Ham F12 medium with 1% (v/v) penicillin/streptomycin, 10% fetal bovine serum (FBS; v/v), 0.25% ascorbic acid (Sigma) and 0.5% insulin-transferrin-selenium (ITS; Thermo Fisher), at a density of 1.0 × 10^6^ per T-75 cell culture flask at 37°C, under an atmospheric oxygen tension of 20% and 5% CO_2_. The remaining half of the cell suspension was seeded in a MSC growth promotion medium (MGPM; alpha modified Eagle’s medium (aMEM), 10% (vol/vol) FBS, 5 ng/mL FGF2 (Sigma)), at a cell density of 1 × 10^6^ cells/T-75 flask and cultured at 37°C with low oxygen tension of 5% O_2_ with 5% CO_2_. Non-adherent cells were removed after 48 h and the medium was changed every 3 days. Colony-forming unit fibroblasts (CFU-Fs) were counted in each culture every 2–3 days. Established cultures of adrenal cells seeded in either a complete medium or MGPM were sub-cultured when 80–90% confluent by enzymatic detachment using TrpLE Express (Invitrogen) and seeded at a density of 1 × 10^6^/75 cm^2^ density. All cell culture reagents were purchased from Gibco (Invitrogen) unless otherwise stated.

### Immunocytochemistry/immunofluorescent staining study

Following first passage of the primary cell culture, cells were re-plated onto sterile coverslips and cultured for a further 24–48 h. Monolayer cells were washed with 1% phosphate buffered saline (PBS) and fixed in 2% paraformaldehyde for 10 min at room temperature. Autofluorescence was quenched with 1 M ammonia chloride for 5 min. Antigen retrieval was carried out where appropriate by incubating the cells in 0.1% Triton X-100 for 3 min at room temperature followed by 3 washes with PBS. Antigenic blocking was performed with 1% bovine serum albumin (BSA) and 5% goat serum for 30 min at room temperature. This was followed by incubation with the primary antibodies in a humidified chamber overnight at 4°C. Cells were then washed with PBS and incubated with appropriate secondary antibodies at room temperature for 1 h in the darkness. Cells were then washed and mounted with Vectashield containing 4ʹ,6-diamidino-2-phenyl-indole (DAPI) (Vector Laboratories, Peterborough, UK). The cells were visualised immediately using an AxioImager Z1 microscope (Zeiss fluorescent microscope with ApoTome attachment). A negative control, without primary antibodies, was used to identify background autofluorescence and also to check for nonspecific binding. The isotype-matched species antibodies (Santa Cruz Biotechnology) were also used as IgG isotype controls to check for isotype-related background staining. The dilution and manufacturer of the primary and secondary antibodies are detailed in Supplementary Table 1 (see section on supplementary data given at the end of this article).

### Fluorescence-activated cell sorting (FACS)

Flow cytometry was used to characterise the immunophenotypic profile of primary human adrenocortical cells. A set of positive and negative cell-surface markers were used to define MSCs, as described by the position paper of the International Society for Cellular Therapy (ISCT) (15). The haematopoetic MSCs isolated from a post-trauma knee joint aspirate were used as a positive control (24). Following the first and second passage for cells cultured in CM and MGPM respectively, adherent cells were detached and re-suspended in a fluorescence-activated cell sorting (FACS) buffer (PBS with 0.1% (vol/vol) BSA) at a concentration of 5 × 10^6^ cells/mL. These cells were filtered through a 100 µm nylon cell strainer and transferred to sterile microfuge tubes (1 × 10^6^ cells/200 µL/tube). For indirect staining, the single-cell suspensions were incubated at 4°C for 1 h with appropriate unconjugated primary antibodies or isotype-match control antibodies. Cells were washed and incubated with appropriate secondary antibodies at 4°C in the darkness for 40 min. They were then washed twice with cold PBS and re-suspended in a 500 µL FACS buffer for immediate analysis. For direct flow cytometry, single-cell suspensions were incubated with fluorochrome-conjugated primary antibodies on ice and in the dark for 40 min. They were washed twice with cold PBS and re-suspended in 500 µL of FACS buffer for immediate analysis. For intracellular staining with anti-GLI1 and DAX1 antibodies, 100 µL of a fixation medium from the Fix and Perm cell permeabilisation kit (Invitrogen) were added to 100 µL of single-cell suspension following detachment of monolayer cells. These cells were incubated at room temperature for 15 min and washed once with 1 mL of PBS. 100 µL of permeabilisation medium and the appropriate unconjugated antibody or the corresponding isotype control were added. Cells were incubated for 20 min at room temperature and washed once with 1 mL of PBS. The cell suspensions were incubated with secondary antibodies on ice and in the darkness for 40 min. Cells were then washed twice and re-suspended in a FACS buffer for immediate analysis.

The controls comprised unstained cells, cells incubated with secondary antibodies only and isotype-matched species antibodies as IgG control. The dilution and manufacture of the antibodies and fluorochromes used are detailed in Supplementary Table 2. Data analysis was performed using BD FACSDiva software, version 8 (BD, Oxford, UK) and Venturi One, version 2 software.

### *In vitro* MSC multilineage differentiation capacity

Adrenal cortex-derived cells cultured in MGPM and CM were assessed for osteogenic, adipogenic and chondrogenic differentiation capacity following the second passage. All images of the stained monolayer cells were acquired using the Nikon Digital Sight–DSFi1 camera and Nikon NIS-Elements D software (Nikon Metrology, UK).

#### Osteogenic differentiation

Cells were seeded at 2.5 × 10^4^ cells/mL/well in a 12-well plate in a 1:1 DMEM:Ham F12 medium enriched with 10% FBS. Cells were allowed to attach overnight and the medium was replaced with StemPro osteogenesis differentiation medium (Invitrogen) the next day. The medium was changed every 3 days and the cells were maintained in an osteogenesis differentiation medium for 21 days. The cell monolayer was then fixed in 2% (v/v) paraformaldehyde prior to staining with Alizarin Red S (ARS), for detection of calcium-rich deposits. In each well of a 12-well plate, cells were incubated with 1 mL of 1% Alizarin Red S solution (v/v) at room temperature for 30 min. Cells were then washed, air-dried and images acquired.

#### Adipogenic differentiation

Following plating on a 12-well plate at a density of 10 × 10^4^ cells/mL/well in a DMEM:Ham F12 medium enriched with 10% (vol /vol) FBS, cells were incubated overnight and induced with StemPro adipogenesis differentiation medium (Invitrogen) the next day. The medium was renewed every 3 days and cells were maintained in the adipogenic medium for 21 days. The cell monolayer was fixed in 2% (v/v) paraformaldehyde at room temperature for 10 min, before staining with Oil Red O for triglyceride or lipid vacuoles. Paraformaldehyde was removed and the monolayers were washed twice with ddH_2_O. The cells were then washed with 60% (vol/vol) isopropanol for 5 min at room temperature, followed by incubation with 1 mL of Oil Red O (Sigma Aldrich) solution for triglyceride or lipid vacuoles at room temperature for 15 min. The cell monolayer was washed 4 times and images acquired.

#### Chondrogenic differentiation

A pellet culture system was used to assess chondrogenic differentiation ability. Following second passage, 2 × 10^6^ adrenocortical cells were re-suspended in 4 mL of StemPro chondrogenic differentiation medium (Invitrogen) to a final cell density of 5 × 10^5^ cells/pellet. 1 mL aliquots were transferred to sterile 15 mL polypropylene conical tubes and centrifuged to the bottom. The tubes were loosely capped to allow for gas exchange. The cell pellets were incubated in a low oxygen tension of 5% O_2_, hypoxic incubator. The chondrogenic medium was changed once every 3 days. Safranin O staining was carried out to characterise the formation of glycosaminoglycans within the cell pellet following 21 days of chondrogenic induction. The cell pellet was embedded in paraffin and processed via a machine processor. Cell pellet sections embedded in wax were then deparaffinised through Histoclear (4 × 10 min), rehydrated through 100% ethanol and deionised water. The sections were then stained in Harris’ haematoxylin for 4 min, de-stained in acid-alcohol for 10 s and rinsed in deionised water. This was followed by staining with 0.02% aqueous fast green (FCF) (Sigma Aldrich) for 3 min, rinsing in 1% acetic acid for 30 s, then staining with 0.1% aqueous Safranin O (Sigma Aldrich) for 5 min. The slides were rinsed thoroughly in deionised water and dehydrated with 100% ethanol and Histoclear before being mounted using a DePEX mounting medium (Leica Biosystem) and visualised using the Nikon Digital Sight–DSFi1 camera and Nikon NIS-Elements D software (Nikon Metrology, UK).

### RNA extraction and reverse transcription

Total RNA was extracted from cultured cells using a PureLink RNA Mini kit (Thermo Fisher), according to the manufacturer’s instructions. Briefly, cells from a single flask (T25 or T-75) were collected and disrupted with lysis buffer and 1% Gibco 2-Mercaptoethanol (Thermo Fisher). The lysate was then transferred to a 1.5 mL RNase-free tube and passed through an 18-gauge needle multiple times for homogenisation. The samples were then transferred to a nucleic acid-binding spin cartridge with a collection tube provided by the kit for subsequent processes of binding, washing and elution accordingly. RNA purity was assessed using a NanoDrop ND-1000 spectrophotometer. Samples with an OD^260/280^ ratio of less than 1.8 or more than 2.2 were rejected. RNA sample integrity was checked with 1% agarose gel electrophoresis for discrete 18s and 28s ribosomal RNA gel bands. The extracted RNA was then subjected to DNase treatment with a Precision DNase kit (Primerdesign, Southampton, UK) to remove genomic DNA contaminations, according to manufacturer’s recommendations. Purified RNA was stored at −20°C and converted to cDNA within 1 month. 1 µg of RNA was reverse transcribed with a Tetro cDNA Synthesis Kit (Bioline, London, UK), with resulting cDNA working concentration of 50 ng/μL. 1 µg of purified RNA was mixed with 1 µL of oligodT_(8)_ and 10 mM dNTP and DEPC-treated water and incubated at 65°C for 10 min. The reaction mix was put on ice for 2 min before being mixed with a 5× RT buffer, RNase inhibitor and reverse transcriptase (200 units). The sample mixtures were then incubated at 45°C for 60 min and chilled on ice at the end of the reaction. Reverse transcription reactions were conducted in duplicates per sample.

### Gene expression profile with q-PCR TaqMan assay

The mRNA expression of pluripotency stem cell markers was studied using q-PCR with QuantStudio 7 (Life Technologies). 50 ng cDNA (1 µL) was amplified with TaqMan gene expression assays (1 µL) (Applied Biosystem) using TaqMan Mastermix (5 µL) (Applied Biosystem) and sterile, distilled, nuclease-free water in a 10 µL reaction volume. The reaction conditions were as follows: 95°C for 10 min, followed by 40 cycles of 95°C for 15 s, then 60°C for 1 min for annealing/extension. Gene expression was quantified by determining the cycle threshold (Ct), which is the number of PCR cycles required for the fluorescence to become significantly higher than the background. Gene expression was normalised against expression of GUSB (endogenous control) and calculated using 2^−ΔΔCt^ method. GUSB was selected as the endogenous control as it showed stable expression across all samples irrespective of culture condition or passage numbers, compared to other 32 candidate genes, using TaqMan array human endogenous controls plate (Thermo Fisher). Each run of experiments were performed in triplicates. A positive control (H295R or MC7 cell line) was included on each experiment. No-template controls and RNA were also included with each run to check for gDNA contamination. Results were first analysed with QuantStudio 7 software. Target mRNA expression relative to GUSB was calculated using the comparative Ct method with Microsoft Excel and GraphPad Prism 6. Ct values above 39 were considered negative for expression. Mean gene expression (2^−ΔΔCt^) was calculated between three independent experiments and expressed with the standard error of the mean (s.e.m.). The details of the TaqMan gene expression assays can be found on Supplementary Table 3.

### Calculations of react ion efficiency and fold-change of gene expression

The efficiency of each TaqMan gene expression assay was calculated by plotting the average triplicate Ct value (*y*-axis) against the logarithm of the amount of cDNA (*x*-axis). A 10-fold, 5-fold or a 2-fold dilution series were used for each gene expression assay. The assay or primer efficiency was determined by calculating the gradient of the slope between the average Ct value and the amount of cDNA, using linear regression analysis on GraphPad. The slope gradient should be approximately −3.32, equivalents to 100% efficiency. The primer efficiency (*E*) was calculated using the following formula *E* = 10^(−1/slope)^ − 1 × 100. The amplification curve determining primary efficiency is available on Supplementary Fig. 1. All the TaqMan gene expression assays managed to establish a slope gradient between −3.22 and −3.60, corresponding to primer amplification efficiency of 90–105%.

All q-PCR experiments were performed in duplicates on the same cDNA samples on biological triplicates. Relative gene expression was corrected using the GUSB housekeeping gene. The mean fold-change expression (2^−ΔΔCt^) was calculated for mRNA expression in cells harvested in MGPM, in relation to cells cultured in CM, which were used as the control.

### Statistical analysis

Statistical analysis was performed using paired Student’s *t* test or Wilcoxon singed-rank test. Data were analysed using Microsoft Excel, GraphPad, version 6 and Minitab version 16. All the data were expressed as the mean of 3 sample replicates (*n* = 3) with the standard error of the mean (s.e.m.). Statistical analysis was deemed significant when *P* < 0.05.

## Results

### Morphology of human adrenal cortex-derived primary cells

**Figure 1:**
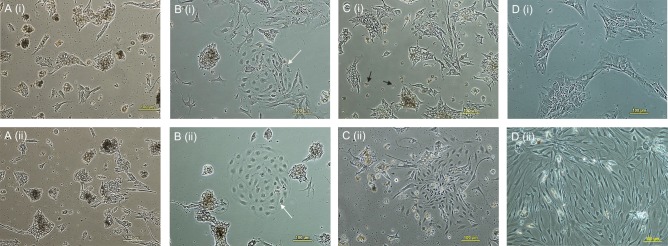
Representative images of primary cell cultures seeded in CM (i; upper panel) or MGPM (ii; lower panel), on day 2 (A), day 3 (B), day 5 (C) and passage 3 (D). (A) (i) and (ii) the primary cells seeded in CM or MGPM were predominantly comprised large adrenocortical cells with polygonal appearance and light-refractive core. (B) (i) and (ii) fibroblast-like colony-forming unit (white closed-head arrow) was formed in CM and MGPM respectively. (C) (i) and (ii) primary cells seeded in MGPM were gradually replaced by fibroblast-like cells (ii), whereas adherent cells in CM (i) were predominantly occupied by large polygonal adrenocortical cells with light-refractive lipid core and fragments that protruded from the cells (black closed-head arrow). (D) (i) and (ii) a homogenous cell population comprised fibroblast-like cells continued to thrive in MGPM after third passage (ii). On the contrary, cell population in CM (i) reached senescence following passage 3. (Magnification ×100.)

Adherent cells isolated from the adrenal cortex comprised highly heterogeneous populations in primary culture, predominantly large polygonal adrenocortical cells with light-refractive core (Fig. 1A (i) and (ii)). However, a relatively homogenous population of smaller, elongated fibroblast-like and spindle-shaped cells were derived after subsequent subcultures in MGPM (Fig. 1D (ii)), which survived long-term culture (up to 11 passages). On the contrary, adherent cells harvested in CM continued to exhibit heterogenous populations on subculture and reached senescence after 3rd passage (Fig. 1D (i)). The colony-forming unit fibroblast-like cells (CFU-F) were seen in the primary culture in either medium but cells seeded in MGPM exhibited a higher number of CFUs (Fig. 1B (i) and (ii)). On average, 4 CFU-Fs per T-75 flask had formed by day 7 after seeding in MGPM, compared to an average of 1.5 CFU-Fs per T-75 flask in CM. The identification of CFU-F formation and the expansion of homogenous cell population with fibroblast-like morphology were in keeping with an MSC-like phenotype.

### Immunophenotyping and immunofluorescent studies

**Figure 2:**
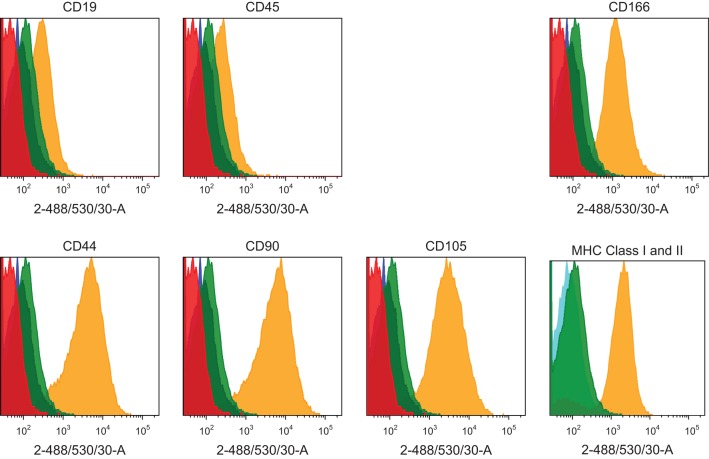
Representative MSC immunophenotype profile of adrenal cortex-derived cells seeded in CM or MGPM. The panel of histograms represent MSC immunophenotypes of cell populations isolated from human adrenal cortex. The adrenocortical cells displayed strong signals towards CD44, CD90, CD105 and CD166 markers but negative for haematopoitic, lymphocytic markers (CD19 and CD45) and HLA-DR (MHC class II). *x*-axes display fluorescent signal intensity and *y*-axes represent cell count. (Red histogram: unstained cells; blue histogram: negative control with fluorochrome-conjugated secondary antibodies only; green histogram: species-specific isotype control; yellow histogram: the staining of cells with antibodies.) The colour codes are applicable to all histograms except graph on MHC class I and II, where blue histogram indicates negative control, yellow and green histograms represent MHC class I (HLA-ABC) and MHC class II (HLA-DR), respectively.

**Figure 3:**
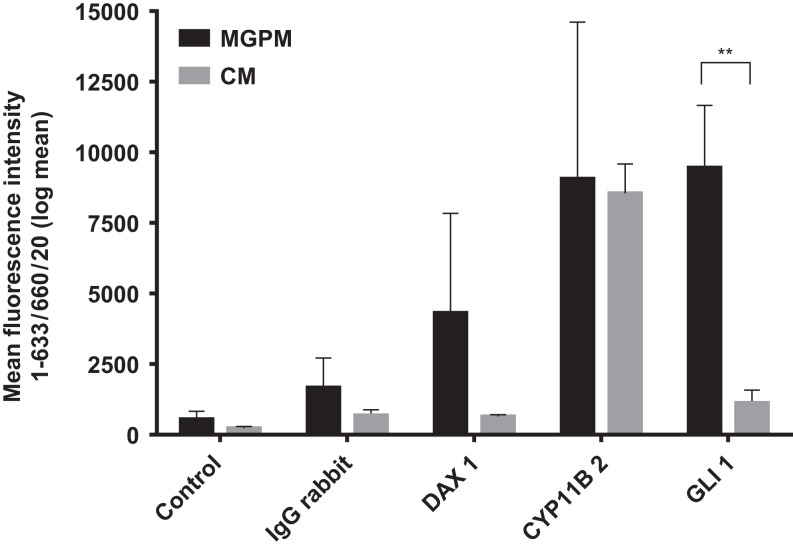
DAX1, GLI1 and CYP11B2 expression among adrenal cortex-derived cell populations cultivated in MGPM vs CM. Cell population harvested in MGPM showed significantly higher GLI1 protein expression (*P* = 0.046; 95% CI for mean differences 391-16274). DAX1 was expressed in cells cultured in MGPM but there was negligible immunostaining in those harvested from CM (NS). No significant difference was demonstrated in CYP11B2 (expression between the cell populations harvested in either medium. *y*-axis represents the signal intensity (mean with s.e.m.).

**Figure 4:**
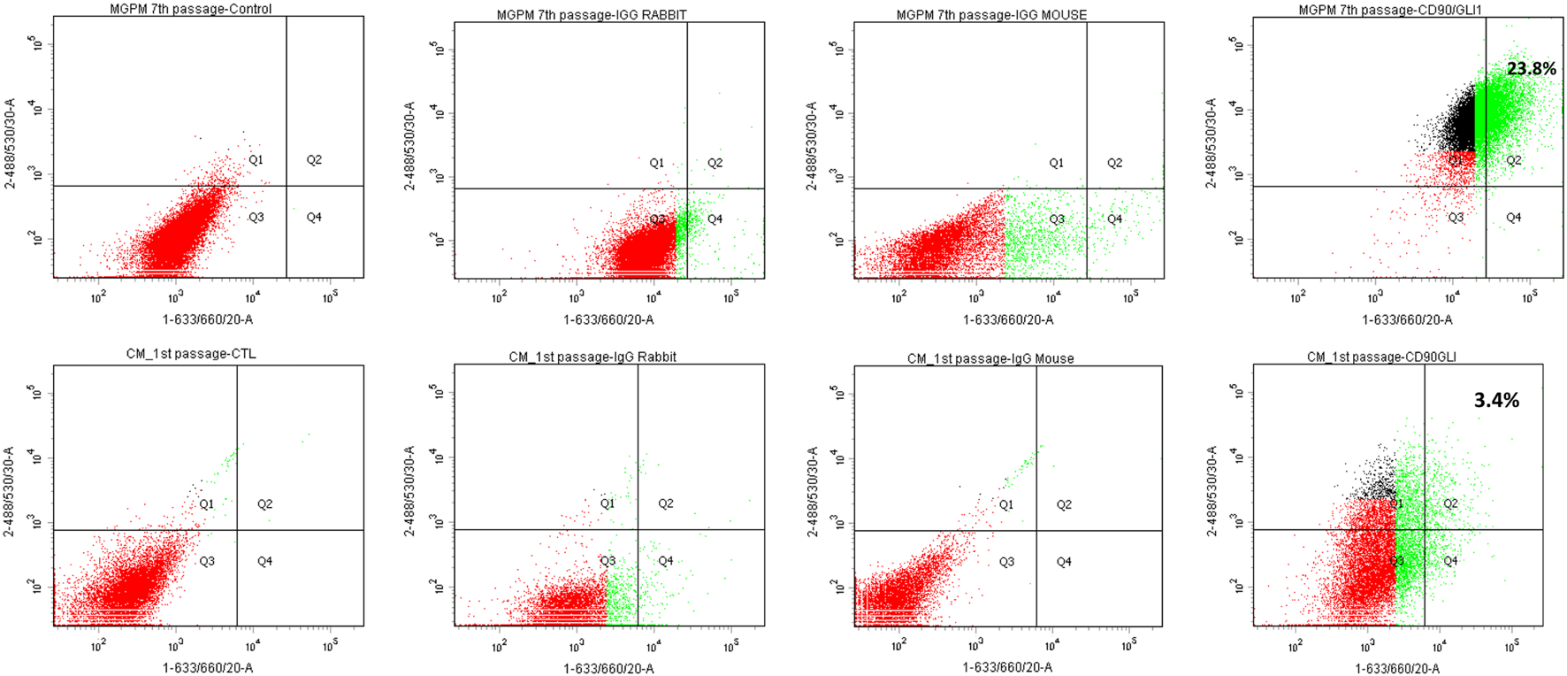
It is a representative dot plot depicting the percentage of cell population (first passage in CM and 7th passage in MGPM) dual-stained with MSC (FITC labelled-488/530/30) and GLI markers (APC-633/660/20). Dual-staining was indicated by the Q2 region, in which >98% of the control and species-specific isotype controls fall outside this area. Approximately 23.8% of the cell population in MGPM co-expressed MSC marker and GLI1 following 7 passages, compared with 3.4% of those cultured in CM after the first passage.

Flow cytometry immunoprofiling demonstrated that cell populations in both MGPM and CM harboured cells expressing mesenchymal markers CD44, CD90, CD105 and CD166 but lacked expression of the haematopoetic and lymphocytic markers: CD19, CD45 and HLA-DR (Fig. 2). The mean percentage of cells positive for all the MSC markers studied, following the first passage was significantly less in those seeded in CM compared to those from MGPM (CM vs MGPM; mean ± s.d.: 57.7 ± 24.6% vs 83.9 ± 14.5%; *P* = 0.006). The cell population harvested from MGPM showed significantly higher GLI1 protein expression, as shown by the higher mean fluorescence intensity (log mean 9456) compared to those seeded in CM (log mean 1123) (Fig. 3; *P* = 0.046; 95% CI for log mean differences 391–16,274). There was a non-significant trend towards a higher mean fluorescence intensity for DAX1 among cells seeded in MGPM compared to CM. Double-staining of adrenal cells also revealed a cell population harvested in MGPM that co-expressed MSC cell-surface markers along with GLI1 (Fig. 4).

**Figure 5:**
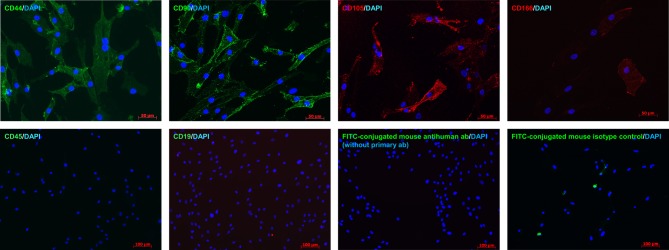
It shows immunolabelling of adrenocortical cells cultured in MGPM for MSC surface markers. Cells from both media showed positive staining for CD 44, CD90, CD105 and CD166 (magnification ×200) but negative staining for CD19 and CD45 markers. (Magnification ×100.)

**Figure 6:**
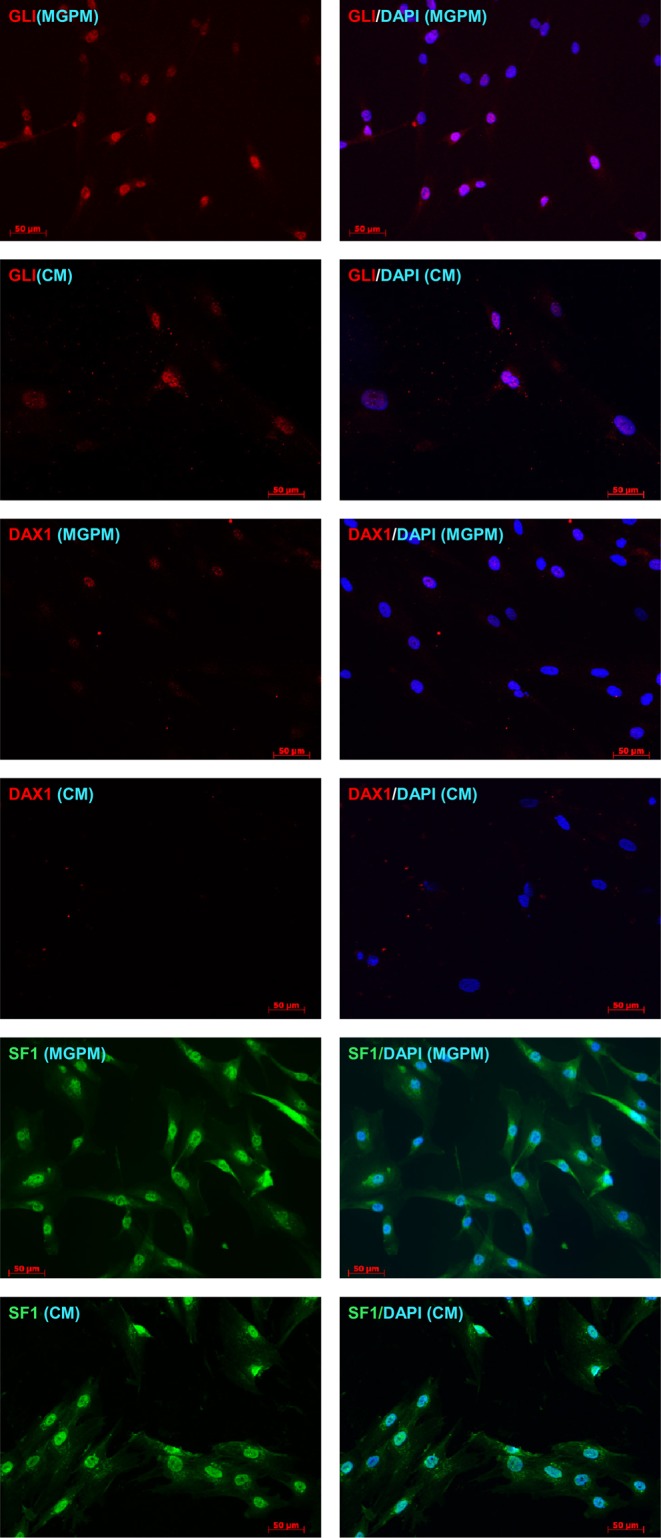
Adrenocortical cells cultured in MGPM and CM were stained for expression of adrenal-specific (SF1) and stem cell morphogenic signalling markers (GLI1, DAX1) and examined under fluorescence microscopy. Nuclei were stained with DAPI (blue, right hand column). Cells cultured in MGPM showed positive staining for SF1, GLI1 and DAX1. The cell population seeded in CM demonstrated strong SF1 expression but weak GLI with negative DAX1 staining. (Magnification ×200.)

**Figure 7:**
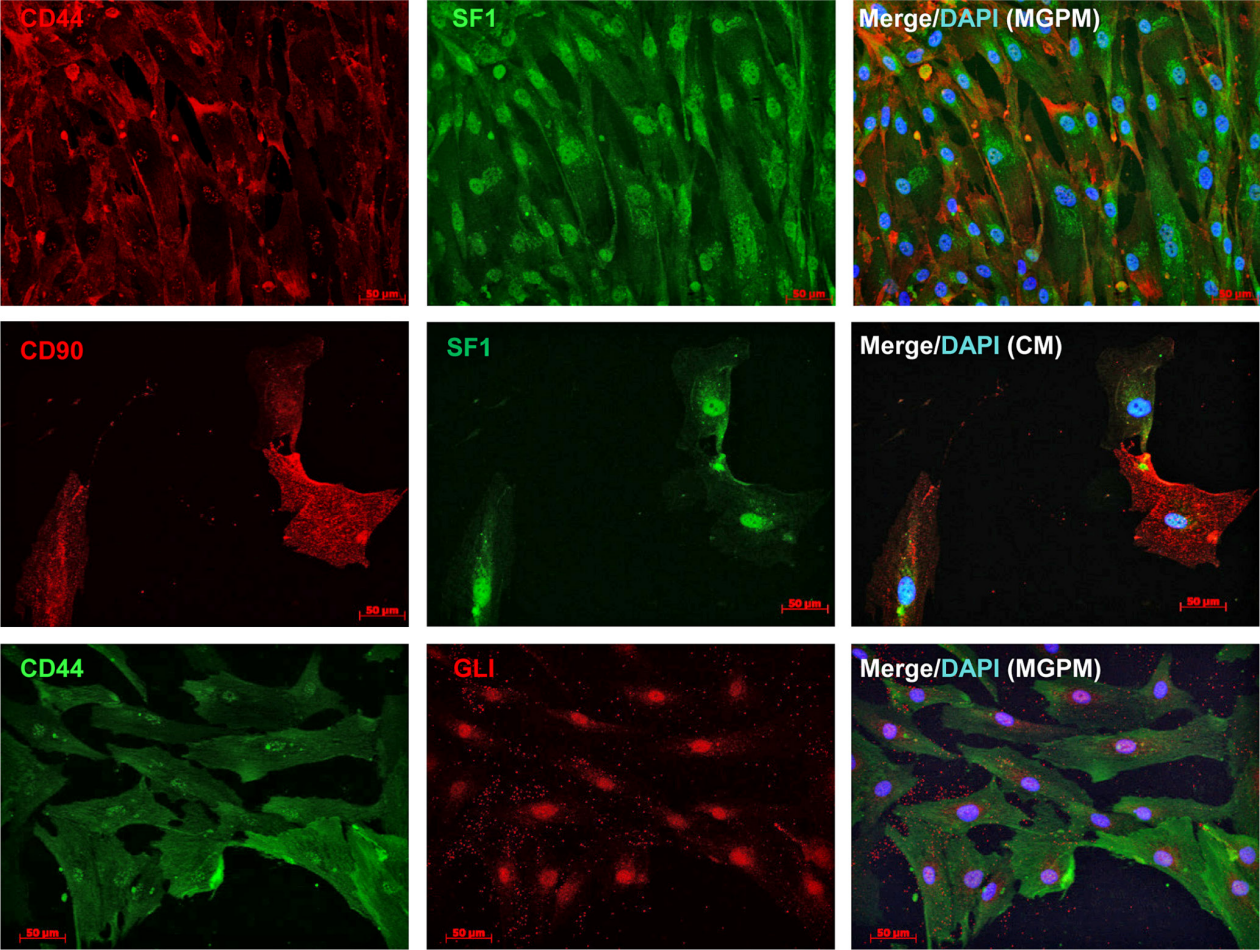
Double-immunostaining of adrenal cortex-derived cells with MSC and SF1/GLI markers. SF1 co-localised with MSC markers in cell populations seeded in either MGPM or CM, as indicated by the membranous staining of CD44/CD90 markers and positive nuclear staining for SF1 markers. Cell population cultured in MGPM also co-expressed MSC and GLI1 marker.

The flow cytometry results were then verified by immunofluorescent studies. Cell populations from both culture conditions showed strong membranous staining towards CD44, CD90, CD105 and CD166 markers but negative staining for CD19 and CD45 antibodies, in keeping with the flow cytometry immunoprofile (Fig. 5). However, cells harvested from CM lost their positive staining towards CD 105/CD166 with only weak CD44/CD90 staining following the second and third subcultures. In contrast, cell population in MGPM showed persistent positive staining towards all MSC markers on every subsequent subcultures. Cell populations from both CM and MGPM also showed positive nuclear staining for the adrenal-specific marker SF1, and the morphogenic signalling GLI1 marker (Fig. 6). GLI1 was observed to be more stably expressed among the cell population from MGPM compared to those from CM. Positive GLI1 staining was found in two of the three adrenal samples seeded in CM, following first passage only. On the other hand, weak positive nuclear staining towards DAX1 was limited to primary cells harvested from MGPM at first passage only, 5 days following subculture. Double-immunostaining revealed that MSC markers co-localised with SF1 and GLI1 antigens (Fig. 7).

### Multilineage differentiation capacity

**Figure 8:**
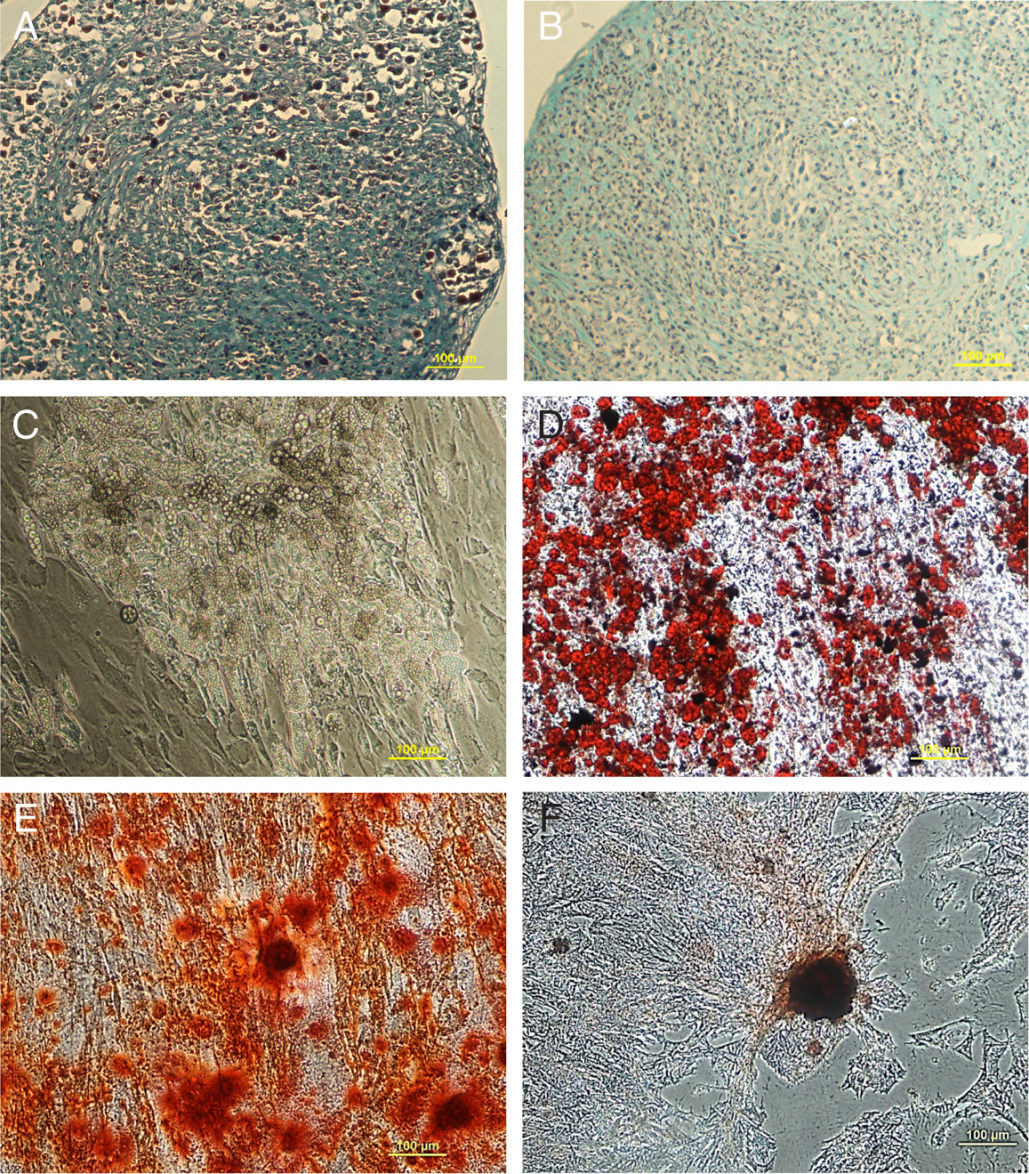
It demonstrates the multilineage differentiation capacity of adrenal cortex-derived cells. Adrenal cells cultured in MGPM were processed into cell pellets and induced with chondrogenic differentiation medium for 21 days. One of three cell pellets (from 3 individual patients) demonstrated weak staining with Safranin O solution, signifying the presence of GAG (A) but two of the remaining 2 showed negative staining (B). Only cells seeded in MGPM survived adipogenic differentiation medium and differentiated into adipogenic cells in abundance, as indicated by cellular accumulation of lipid-rich vacuoles (C), which stained with Oil Red O (D). Cell population seeded in either CM or MGPM (E) or CM (F) differentiated into osteogenic lineage at day-21 with enhancement of alkaline phosphatase activity as shown by the positive staining for Alizarin S.

Cell populations harvested in both CM and MGPM were examined for their multilineage differentiation capacity. MSC-like adrenocortical cells harvested in MGPM showed strong osteogenic and adipogenic differentiation capacity. In contrast, they exhibited poor chondrogenic capacity with a weak and variable degree of differentiation to the chondrogenic lineage; only 1 of the 3 individual adrenal tissue culture demonstrated weak Safranin O staining, indicating the presence of GAGs within the pellet matrix (Fig. 8A and B).

Osteogenic differentiation was seen in cell populations from both media, as demonstrated by positive Alizarin Red S staining indicating the presence of extracellular calcium deposits (Fig. 8C and D). However, osteogenic induction was significantly weaker in cell populations harvested from CM and the same cell population failed to survive the induction medium for adipogenesis and chondrogenesis. Morphologic changes consistent with adipogenesis were observed as early as day-14, as shown by the transformation of spindle-shape fibroblast-like MSC cells to lipid-laden adipocyte-like cells (Fig. 8E). The presence of lipid vacuoles was confirmed with positive Oil Red O staining, consistent with adipogenesis (Fig. 8F).

### Transcriptional profile with RT-q-PCR

**Figure 9:**
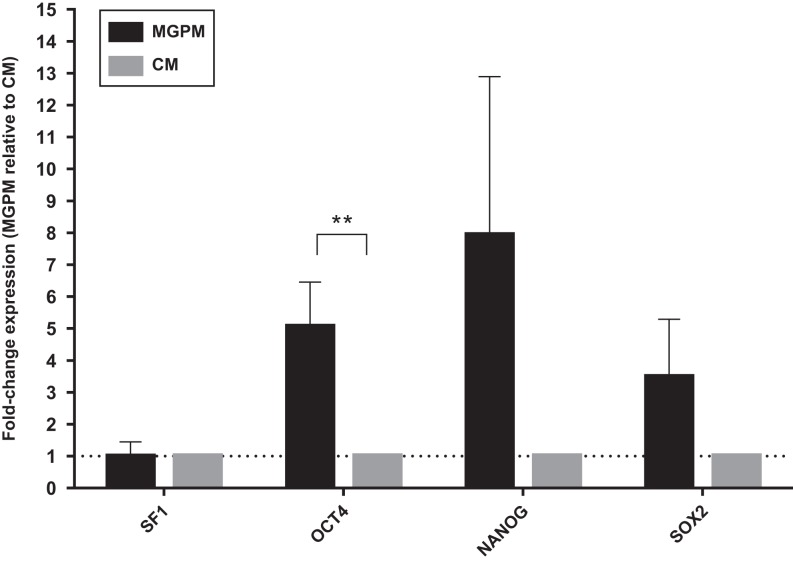
Adrenal cortex-derived cells cultured in CM and MGPM respectively were analysed by q-PCR for the endogenous expression of pluripotency markers (NANOG, OCT4 and SOX2) and steroidogenic factor 1 marker. Error bars represent standard error of means for cell population from 3 individual patient-derived cultures. Fold changes of gene expression were calculated with the 2^−ΔΔCT^ method, using *GUSB* as the house keeping gene. Results were shown as mean ± s.e.m. from triplicates (*n* = 3), using paired *T* test. Differences in gene expression OCT4 was statistically significant in cell population harvested from MGPM (**P-value <0.05) relative to the cell population cultured in CM.

Cell populations from both CM and MGPM were analysed by RT-q-PCR for the endogenous expression of pluripotent markers (NANOG, SOX2 and OCT4) (Fig. 9). Gene expression of pluripotency markers was higher in cell population harvested from MGPM. OCT4 expression was significantly higher in cells harvested in MGPM (*P* = 0.024, 95% CI for mean fold-change difference: 1.07–8.71).

## Discussion

This study demonstrates that the human adrenal cortex harbours a mesenchymal stromal/stem cell-like population, which exhibits the phenotypic signature of both MSCs and adrenocortical cells. This cell population maintained their proliferative capacity in the MSC growth promotion medium (MGPM) under hypoxic conditions and expressed pluripotency markers. These features suggest their potential role as the previously uncharacterised multipotent human adrenocortical progenitor cells. They have a high proliferative capacity and differentiated well along osteogenic and adipogenic lineages, but showed only limited chondrogenic differentiation. Distinct features have been observed among MSCs isolated from different organs, with respect to their immunophenotype and secreted cytokine profile (25, 26). Hence, the limited chondrogenic differentiation could be due to the lack of innate chondrogenic potential or related to the *in vitro* cell expansion techniques.

It has been postulated that ACSCs potentially originate from capsular or subcapsular undifferentiated cells expressing GLI and SF1, whereas DAX1 is the transcription factor for maintaining the ‘stemness’ of adrenal stem/progenitor cells, by turning off the cellular differentiation capacity in the absence of ACTH (27). In this study, primary cells harvested in MGPM, but not in CM, strongly expressed GLI with weak DAX1 expression, indicating that ‘stemness’ was enhanced among the cell population in MGPM. This appears consistent with their higher proliferative capacity and potential for long-term culture. GLI1 is a transcription factor in the sonic hedgehog pathway, an essential signalling pathway in rodent ACSC (28). The MSC-like cells derived from adrenal cortex also co-expressed GLI and SF1 markers, enhancing the evidence that they were predominantly adrenal progenitor cells. However, this study had a few limitations. Firstly, primary adrenal cortex-derived cells comprised highly heterogeneous cell populations in line with the zonal differentiation of the gland and hence it is unlikely that there will be a single defining molecular characteristic of these derived cell populations. Nevertheless, following isolation, hypoxic culture and several passages in mesenchymal growth media, our work is consistent with a homogenous population of MSC-like cells existing within human adult adrenal cortex. These MSC-like adrenocortical cells could either represent a static population of adrenocortical progenitors or non-ACSCs that co-exist in the stem cell niche with MSC properties that have migrated from other tissues. Furthermore, as this is the first study to isolate MSC-like cells from the human adrenal cortex, there are no established protocols for this. Therefore, different isolation and culture techniques, such as the use of a digestive enzyme like the recombinant fungal trypsin-like protease used in this study, might affect MSC function or differentiation capacity by nonspecific degradation.

The identification of a MSC-like population in the adrenal cortex has contributed towards our understanding of adrenal plasticity. Although these adrenal cortex-derived MSC-like cells are yet to be proven as ACSC, they have been found to exhibit features consistent with progenitor cells. Similar to non-bone marrow-derived MSC isolated from other tissues, the exact anatomical location of these MSC-like cells in intact human adrenal has not been defined. Many studies suggest that this group of cells resides on the luminal surface of endothelial cells in the perivascular niche, namely pericytes or adventitial cells (29, 30). They have also been shown to home in on injured or inflamed tissues, with anti-inflammatory properties. They promote tissue repair through the production of trophic factors, which reduce inflammation and facilitate functional recovery of the damaged cells (31, 32, 33). Promising results have been demonstrated in experimental murine models of acute tissue injury and autoimmunity, such as autoimmune encephalomyelitis, glomerulonephritis and acute fulminant liver failure (34, 35, 36). Interestingly, MSCs seem to demonstrate plasticity and to have the capacity to adapt functionally according to their environment, as MSC therapy has been shown to work in active autoimmune encephalitis but not during remission (37, 38). A few phase I clinical trials have also demonstrated clinical safety and the feasibility of MSC therapy in inflammatory bowel disease (39), liver cirrhosis (40) and myocardial infarction (41). In view of these encouraging preliminary results, the discovery of MSC-like cells in the human adrenal cortex should prompt further investigation with respect to their *in vivo* identity and biological function, to form a potentially therapeutic tool for adrenal diseases.

MSC therapy certainly appears to have attractive therapeutic potential in autoimmune diseases. Experimental treatments of type 1 diabetes using MSC therapy in murine models have already shown some promising results. These studies demonstrated an increase in the number of pancreatic insulin-producing beta-cells with suppression of auto-reactive T lymphocytes and inflammatory dendritic cells, leading to long-term reversal of hyperglycaemia (32, 42, 43). With this in mind, an understanding of the cell biology of adrenal-derived MSCs will be essential to the regenerative medicine approach in AAD as well as other autoimmune endocrine diseases (i.e. Graves’ orbitopathy). Furthermore, these cells are strongly implicated in adrenocortical cancer and improved knowledge of their characteristics has the potential to inform future cancer therapy.

The anatomical niche of adrenal-MSCs at the tissue level, as well as the genomic and proteomic properties of this cell population, also warrants detailed investigation, potentially by q-PCR and proteomic studies with single-cell analysis. A comprehensive high-throughput screening of the actions of various morphogenic and transcription factors during differentiation of MSC to steroidogenic cells will also help identify the agents or factors essential for adrenal self-renewal and long-term steroidogenesis. Finally, developing a method for differentiating MSCs into steroidogenic cells, in which the bone marrow or adipose tissues are a rich source for MSCs, could potentially lead to curative cell therapy for AAD.

## Supplementary Material

Supporting Figure 1

Supporting Table 1

Supporting Table 2

Supporting Table 3

## Declaration of interest

The authors have no conflict of interest that could be perceived as prejudicing the impartiality of the research reported.

## Funding

This work was supported by grant G09000001 from the Medical Research Council, UK.

## Author contribution statement

Earn Gan: Conception and design, data collection, analysis and interpretation, manuscript writing. Wendy Robson and Peter Murphy: Provision of study materials and obtaining informed consent. Robert Pickard: Provision of study material. Simon Pearce: Conception and design, data analysis and interpretation, final approval of manuscript. Rachel Oldershaw: Study design, data analysis and interpretation and final approval of manuscript.
