# Supplementary material for: Isolation of a multipotent mesenchymal stem cell-like population from human adrenal cortex
Source: Endocr Connect. 2018 Apr 5;7(5):617–29. doi: 10.1530/EC-18-0067 (PMC5919938; doi:10.1530/EC-18-0067)

Figure 1. PCR efficiency curve for Taqman gene expression assays used in this study. The y-axis indicates the average triplicate Ct value for each assay and the x-axis represents the logarithm of the amount of cDNA. A 10-fold, 5-fold or a 2-fold dilution series were used for each gene expression assay. All the Taqman gene expression assays established a slope gradient between -3.22 to -3.60, corresponding to primer amplification efficiency of 90-105%.

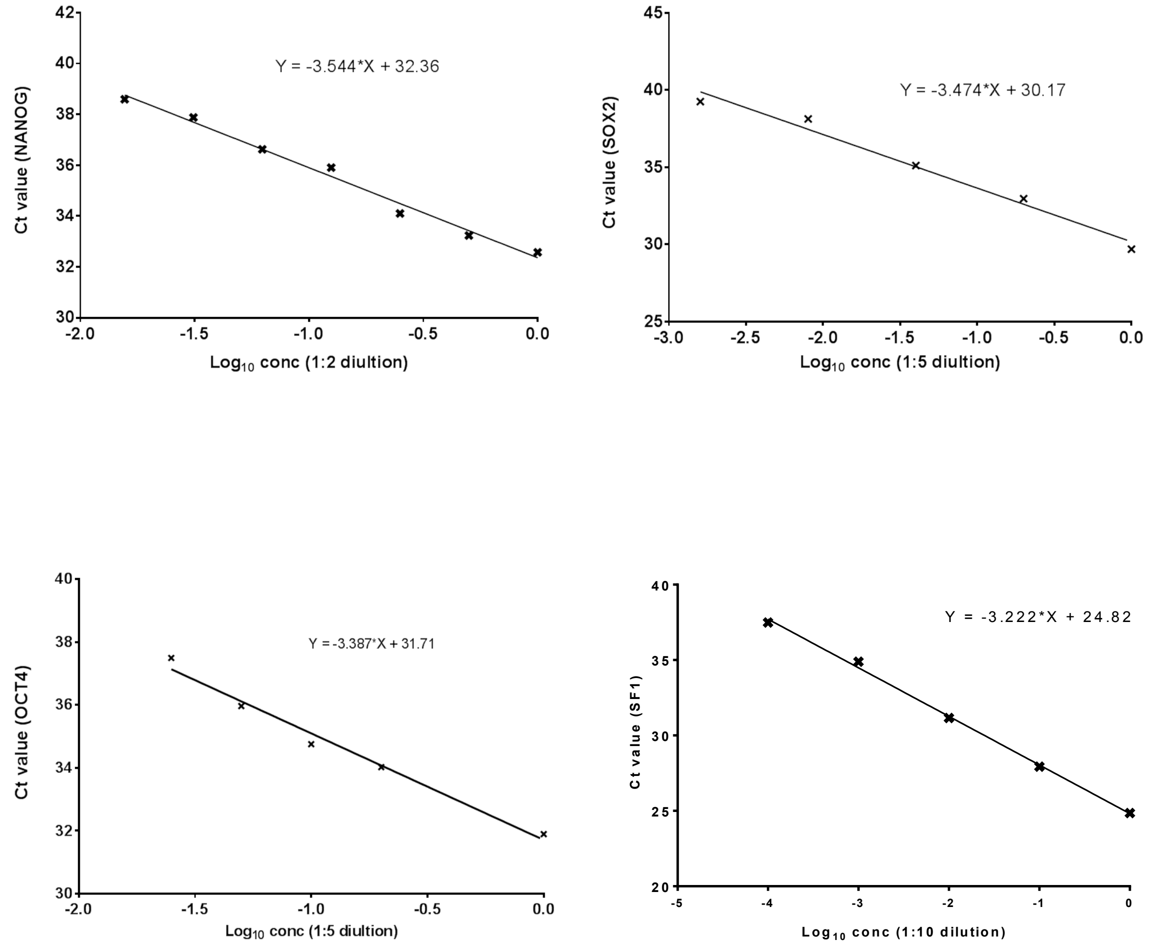

Supplement: Supporting Figure 1 [file ec-7-617-s001.pdf]
