# Supplementary material for: Isolation of a multipotent mesenchymal stem cell-like population from human adrenal cortex
Source: Endocr Connect. 2018 Apr 5;7(5):617–29. doi: 10.1530/EC-18-0067 (PMC5919938; doi:10.1530/EC-18-0067)
Supplement: Supporting Table 1 [file ec-7-617-t001.pdf]

Supplementary 1

Table 1. Primary and secondary antibodies used for immunofluorescence

**Primary antibody**	**Source**	**Dilution (final concentration)**	**Catalogue number**
**GLI rabbit IgG**	Santa Cruz Biotechnology	1:100 (2µg/ml)	SC20687
**SF1 goat IgG**	Santa Cruz Biotechnology	1:100 (2µg/ml)	SC10976
**DAX1 rabbit IgG**	Abcam	1:200 (2µg/ml)	Ab97369
**MSC antibody panel including CD19, CD44, CD45, CD90, CD105, CD106, CD146, CD166**	R&D system	1:40 (2.5µg/ml)	SC017

**Secondary antibody**	**Source**	**Dilution(final concentration)**	**Catalogue number**
**Bovine anti-goat IgG-FITC**	Santa Cruz Biotechnology	1:1000 (0.4 µg/ml)	S2348
**Sheep anti-rabbit IgG-Texas Red**	Abcam	1:2000 (1 µg/ml)	Ab6793
**Goat anti-mouse IgG-AF488**	Invitrogen	1:2000 (1 µg/ml)	A-10667
**Donkey anti-mouse IgG-AF 594**	Invitrogen	1:2000 (1 µg/ml)	A-21203
