# Supplementary material for: Isolation of a multipotent mesenchymal stem cell-like population from human adrenal cortex
Source: Endocr Connect. 2018 Apr 5;7(5):617–29. doi: 10.1530/EC-18-0067 (PMC5919938; doi:10.1530/EC-18-0067)
Supplement: Supporting Table 2 [file ec-7-617-t002.pdf]

Table 2. Unconjugated and conjugated primary antibodies used in flow cytometry

Primary antibody	Source	Dilution	Catalogue number
Unconjugated primary antibody			
**DAX1 rabbit IgG**	Abcam	1:10 (40 µg/ml)	Ab97369
**GLI rabbit IgG**	Santa Cruz Biotechnology	1:10 (20 µg/ml)	SC20687
**MSC antibody panel (CD19, CD44, CD45, CD90, CD105, CD106, CD166 mouse IgG)**	R&D system	1:1 (100 µg/ml)	SC017
Conjugated primary antibody			
**MHC I HLA-ABC Antibody, FITC conjugate**	Thermo Scientific	1:1 (100µg/ml)	MA1-80454
**MHC II HLA-DR Antibody, FITC conjugate**	Thermo Scientific	1:1 (100µg/ml)	MA1-19620

Secondary antibody	Source/manufacture	Dilution	Catalogue number
**APC-conjugated goat anti-rabbit IgG**	Santa Cruz Biotechnology	1:100	SC3846
**FITC-conjugated goat anti-mouse IgG**	Sigma Aldrich	1:100	F2653
