# Supplementary material for: Isolation of a multipotent mesenchymal stem cell-like population from human adrenal cortex
Source: Endocr Connect. 2018 Apr 5;7(5):617–29. doi: 10.1530/EC-18-0067 (PMC5919938; doi:10.1530/EC-18-0067)
Supplement: Supporting Table 3 [file ec-7-617-t003.pdf]

Table 3: Taqman gene expression assays

Gene symbol	Gene name	TaqMan assay	Amplicon size
NANOG	Nanog homeobox	Hs04260366_G1	99 bp
SOX2	SRY-box 2	Hs01053049_s1	91 bp
POU5F1 (OCT4)	POU class 5 homeobox 1	Hs00888632_G1	77 bp
NR5A1 (SF1)	Nuclear receptor subfamily 5 group A member 1	Hs00610436_m1	71 bp
GUSB	Glucuronidase beta	Hs9999908_m1	81 bp

Source: https://www.thermofisher.com/order/genome-database/browse/gene expression/keyword
